# The state of PI monotherapy and NRTI-sparing therapy

**DOI:** 10.1186/1758-2652-13-S4-O17

**Published:** 2010-11-08

**Authors:** JR Arribas

**Affiliations:** 1 Hospital La Paz, Consulta Medicina Interna-2, Madrid, Spain

## 

Current consensus about treatment of HIV infection is that HAART must include two nucleoside reverse transcriptase inhibitors plus a third drug (one non-nucleoside reverse transcriptase inhibitor, a boosted protease inhibitor, an integrase strand transfer inhibitor and possibly a CCR5 inhibitor). There is a lot of interest about changing the basic structure of the antiretroviral regimen so we can be able to use nucleoside-sparing regimens. The main reason to support the investigation of NRTI-sparing strategies is the concern about the long-term toxicity of tenofovir and abacavir.

In antiretroviral naïve patients candidates for nucleoside sparing regimens have included a boosted or unboosted protease inhibitor as the backbone drug to which a non-nucleoside reverse transcriptase inhibitor, an integrase inhibitor, a single nucleoside or a CCR5 inhibitor has been added. In patients who have already achieved suppression it is possible that a boosted protease inhibitor used as monotherapy might be all what is needed to maintain suppression. It is reasonable to predict that, compared to triple-drug HAART, the long term toxicity of these single and dual-drug regimens would be lower. Finding the right number of antiretrovirals that offers the optimal balance of long-term efficacy and toxicity is therefore a very important scientific question.

This presentation would review the clinical trials that have explored NRTI sparing strategies for the treatment of antiretroviral naïve patients and also for maintenance of viral suppression. The presentation would highlight the possible benefits associated to NRTI-sparing strategies and the most promising candidates.

